# Polymorphism and Pharmacological Assessment of Carbamazepine

**DOI:** 10.3390/ijms25189835

**Published:** 2024-09-11

**Authors:** Alberto Sá Filho, Jose Luis Rodrigues Martins, Rafael Fernandes Costa, Gustavo Rodrigues Pedrino, Vitor Santos Duarte, Osmar Nascimento Silva, Hamilton Barbosa Napolitano, James Oluwagbamigbe Fajemiroye

**Affiliations:** 1 Graduate Program in Pharmaceutical Sciences, Evangelical University of Goiás, Anapolis 75083-515, GO, Brazil; doutor.alberto@outlook.com (A.S.F.); jose.martins@docente.unievangelica.edu.br (J.L.R.M.); rafael_fcosta@yahoo.com.br (R.F.C.); osmar.silva@catolica.edu.br (O.N.S.); hbnapolitano@gmail.com (H.B.N.); 2 Institute of Biological Sciences, Federal University of Goiás, Goiás 74605-010, GO, Brazil; pedrino@ufg.br; 3 Structural and Theoretical Chemistry Group, State University of Goiás, Anápolis 75083-515, GO, Brazil

**Keywords:** carbamazepine, polymorphism, formulation, cytoprotection, animal models, anticonvulsant

## Abstract

This work provides insight into carbamazepine polymorphs (Forms I, II, III, IV, and V), with reports on the cytoprotective, exploratory, motor, CNS-depressant, and anticonvulsant properties of carbamazepine (CBZ), carbamazepine formulation (CBZ-F), topiramate (TOP), oxcarbazepine (OXC), and diazepam (DZP) in mice. Structural analysis highlighted the significant difference in molecular conformations, which directly influence the physicochemical properties; and density functional theory description provided indications about CBZ reactivity and stability. In addition to neuron viability assessment in vitro, animals were treated orally with vehicle 10 mL/kg, as well as CBZ, CBZ-F, TOP, OXC, and DZP at the dose of 5 mg/kg and exposed to open-field, rotarod, barbiturate sleep induction and pentylenetetrazol (PTZ 70 mg/kg)-induced seizure. The involvement of GABAergic mechanisms in the activity of these drugs was evaluated with the intraperitoneal pretreatment of flumazenil (2 mg/kg). The CBZ, CBZ-F, and TOP mildly preserved neuronal viability. The CBZ-F and the reference AEDs potentiated barbiturate sleep, altered motor activities, and attenuated PTZ-induced convulsion. However, flumazenil pretreatment blocked these effects. Additional preclinical assessments could further establish the promising utility of CBZ-F in clinical settings while expanding the scope of AED formulations and designs.

## 1. Introduction

Epilepsy is one of the most common and disabling chronic neurological disorders, characterized by recurrent seizures, affecting millions of individuals worldwide [1,2]. Carbamazepine (CBZ) remains the most common pharmacological intervention for partial seizures [3]. However, the search for new anticonvulsant drugs (AEDs) or formulations with lower toxicity, better efficacy and stability continues to attract attention [4]. CBZ is an AED with low aqueous solubility and high permeability. It is currently included as a Class II drug in the biopharmaceutics classification system (BCS). Other attributes such as narrow therapeutic index and relatively high variability have also been identified as barriers for CBZ generic substitution and/or product interchangeability [5,6]. The dissolution of CBZ in vivo is, therefore, the limiting step of its absorption, which is generally slower than Class I drugs in the BCS. The formulation and in vivo variables that can affect the profile of dissolution could account for the variability in the absorption of Class II drugs [7]. As the limiting step for drug absorption and bioavailability, dissolution depends on the pH value, ionic strength, volume of gastrointestinal fluids, and the presence of surfactants, among other factors [6].

Polymorphism in CBZ has been frequently documented [8,9,10]. Structural polymorphism is characterized as the possibility of a solid crystallizing into different crystal forms or alternate arrangements of atoms or molecules in the solid, which can result in different physicochemical properties (melting point, solubility, density, stability, reactivity) [11,12,13]. In the literature, five polymorphic forms for CBZ are described: Form I crystallizes in the triclinic crystalline system (P), Form II crystallizes in the trigonal system (R), Forms III and IV crystallize in the monoclinic crystal system (P21/n, C2/c, respectively), while Form V belongs to the orthorhombic crystal system. For a drug with low solubility, such as CBZ, polymorphism, crystalline habit, and particle size are drug attributes that can impact tablets’ dissolution and, consequently, bioavailability in vivo. The risk of bioinequivalence in formulations containing BCS Class II drugs is often high [6,14,15,16]. As a result of the challenges of oral formulation development, including physicochemical properties and a complex internal environment that limits dissolution and absorption in the GI tract, different strategies including solid dispersions [17], cyclodextrin inclusions [18], and nanoscale formulations [19] have been employed. Although CBZ remains important in the treatment of epilepsy [20], its limited solubility and variable bioavailability due to polymorphism have raised significant concerns regarding its consistent therapeutic outcomes, suggesting the need to search for more efficient drugs.

In addition to polymorphism, unclear antiseizure activities and mechanisms of CBZ are of research interest. Understanding the mechanisms of action can facilitate comprehension of the spectrum of biological activity. Hence, these assessments of drugs remain a relevant approach to determining their characterization, application, and limitations in clinical settings. Although voltage-gated sodium channels are reported as the main target of CBZ [21], the scope of its mechanism of action keeps expanding. The hypothesis of imbalanced inhibitory and excitatory neuronal population activity that is associated with seizures [22] supports the upregulation and downregulation of GABAergic and glutamatergic transmission, respectively. In this manner, the blockade of GABAergic transmission with flumazenil (an antagonist of the benzodiazepine-binding site) could explain the attenuation of anticonvulsant properties. In this study, preclinical assessments in vitro and in vivo were considered relevant for further characterizations of CBZ and other drugs. As voltage-gated ion channels or ionotropic γ-aminobutyric acid (GABA) A receptors, α-amino-3-hydroxy-5-methyl-4-isoxazole-propionate and N-methyl-D-aspartate receptors are associated with the mechanisms underlying AEDs effects [1,23]; exploring neuron viability, exploratory and motor activities, barbiturate sleep induction, and anticonvulsant activity could provide additional insights.

Hence, to resolve this critical issue and enrich the pharmacology of AEDs, this study develops a novel carbamazepine formulation (CBZ-F); explores polymorphic variations of the CBZ molecule; and provides data on biological activities of CBZ, CBZ-F, and other first-class AEDs (topiramate, oxcarbamazepine, diazepam). In addition to the structural analysis of different CBZ polymorphs, this manuscript shows that the supramolecular arrangement of the CBZ polymorphs is stabilized by C-H…O and N-H…O interactions with the presence of dimeric interaction (except for Form V). These interactions were confirmed through electron density using Hirshfeld surfaces (HSs), while theoretical calculations indicated the molecular orbitals and regions susceptible to electrophilic/nucleophilic attacks. The structural analysis combined with theoretical calculations allows us to better understand the physical–chemical properties of these polymorphs.

## 2. Results and Discussions

### 2.1. Structural Polymorphism Analysis

The five forms of CBZ found in the literature and present database have the same molecular structures; however, they exhibit different molecular conformations (polymorphs). Forms III and IV crystallize in the monoclinic crystal system but in different space groups, P21/n and C2/c, respectively. Meanwhile, Forms I, II, and V crystallize in the following groups: triclinic (P$\bar{1}$), trigonal (R$\bar{3}$), and orthorhombic (P*bca*), respectively. Form I has four molecules in the asymmetric unit (Z^′^), whereas the other forms have only one independent molecule in Z^′^. Form II has 18 molecules in the unit cell (Z), and Form III has 4 molecules, while Forms I, IV, and V have 8 molecules in Z (Table 1).

**Figure 1:**
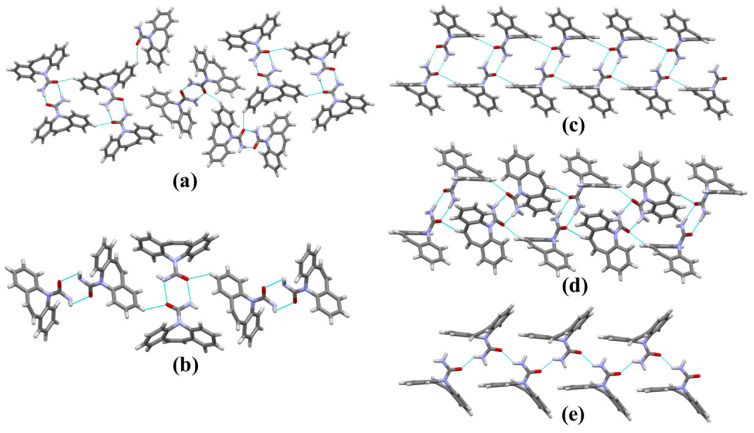
Main interactions of the supramolecular arrangement of the carbamazepine forms. I (**a**), II (**b**), III (**c**), IV (**d**), and V (**e**).

The supramolecular arrangement of forms of CBZ is supported by interactions of the C-H…O and N-H…O type; all forms of carbamazepine, except for Form V, present dimeric interaction (Figure 1). This dimerization in the solid form is related to the donation–reception of electrons between the amide groups that form the N-H…O interaction. The distance of this interaction may be linked to the energy involved and molecular packing. The distances between dimeric interactions for Forms I–IV are similar; the smallest distance between donor and acceptor is 2.847 Å for Form IV (Table 2). The dimers and the short distances of these interactions may contribute to a higher molecular packing density, potentially resulting in increased stability and reduced solubility. The variations in interactions, packaging, and supramolecular arrangement can lead to changes in the solubility of this molecule in water, which is consistent with its irregular absorption among polymorphs.

**Figure 2:**
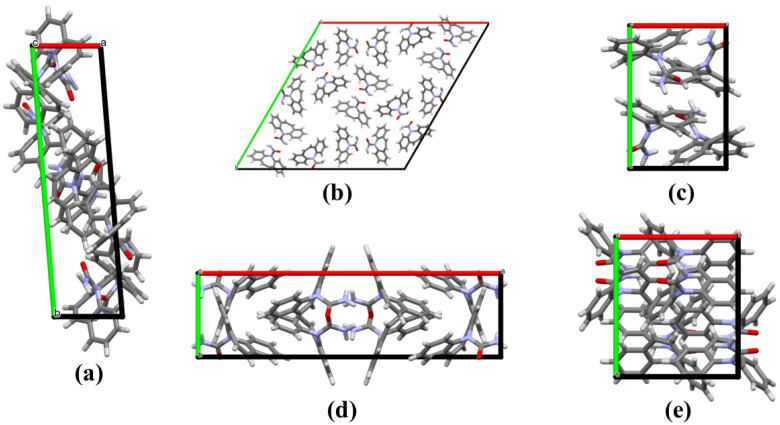
Molecular packing in the unit cell for carbamazepine forms. I (**a**), II (**b**), III (**c**), IV (**d**), and V (**e**).

The quantification of interactions present in the CBZ forms shows similar results; however, the packing in a unit cell of the compound exhibits significant differences (Figure 2). According to the 2D fingerprints, contacts involving hydrogens (H…H) are found in the majority of organic molecules (Form I (47.5%), Form III (53.7%), Form IV (50.6%), and Form V (48%)) (Table 3). The C…H corresponds to C-H…π interactions, and C…C indicates contacts involving aromatics (π…π), which is low for all CBZ forms analyzed. Additionally, O…H indicates classical and non-classical hydrogen interactions such as C-H…O and N-H…O (shown in Figure 1). These O…H interactions for CBZ forms present values around 13%.

The supramolecular arrangement of the analyzed forms of CBZ is determined by a combination of weak and strong interactions, including the presence of a dimer (N-H…O). Notably, Form V of CBZ deviates from the other forms as it lacks solid-state dimerization, which is responsible for the formation of the supramolecular array found in the remaining forms. As hydrogen atoms are absent in the structure of Form 4, it is not feasible to quantify hydrogen-involved interactions using 2D fingerprints.

**Figure 3:**
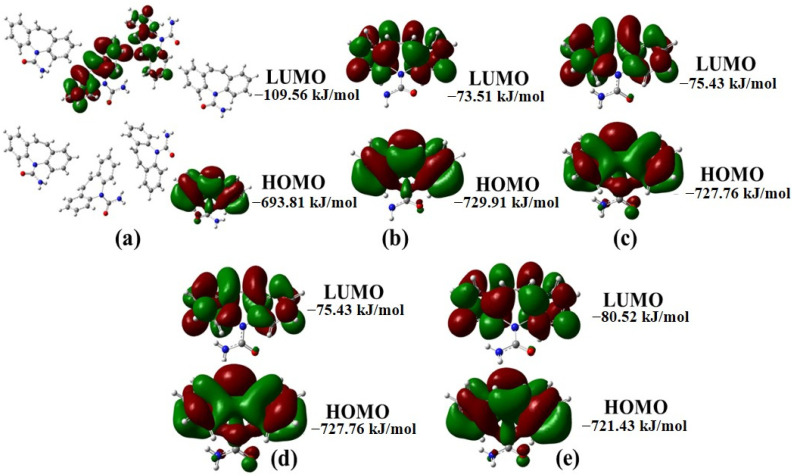
FMO representations for CBZ forms. I (**a**), II (**b**), III (**c**), IV (**d**), and V (**e**).

The distribution of the HOMO orbital is similar among all five molecules, whereas the distribution of the LUMO orbital is consistent for all five molecules except for molecule 1 (Figure 3). The LUMO orbitals of the CBZ forms represent the antibonding orbitals associated with electron affinity (electrophilic behavior), while the HOMO orbitals represent the bonding orbitals associated with ionization potential (nucleophilic behavior). The energy difference between the LUMO and HOMO orbitals, called EGAP, is used as an indicator of some properties of the molecule, such as kinetic stability and chemical reactivity. This information contributes to understanding and predicting the behavior and performance of molecules in diverse chemical systems. The EGAP for a CBZ molecule is 656.40 kJ/mol (II) > 652.33 kJ/mol (III) > 652.33 kJ/mol (IV) > 640.91 kJ/mol (V) > 584.25 kJ/mol (I). These EGAP values suggest that carbamazepine Form II has the highest kinetic stability, while Form I has the lowest stability and highest reactivity.

**Figure 4:**
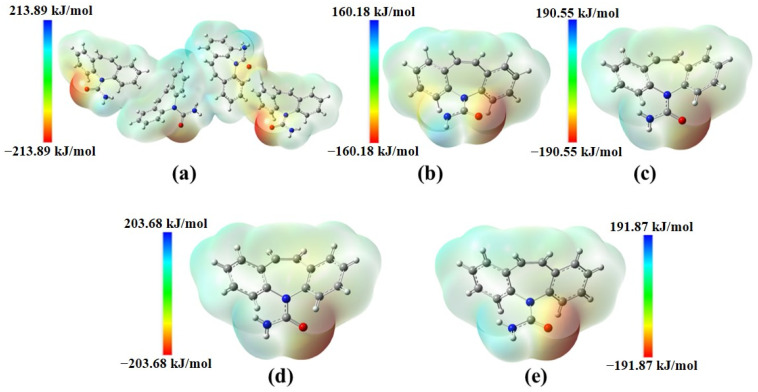
MEP map for CBZ forms. I (**a**), II (**b**), III (**c**), IV (**d**), and V (**e**).

The MEP map suggests that the oxygen atom of the amide group has high electron density, suggesting these regions are more susceptible to electrophilic attacks, whereas the regions with hydrogen atoms were shown to be most susceptible to nucleophilic attack (Figure 4).

CBZ is a widely used antiepileptic drug known for its polymorphic nature, which refers to the ability of a compound to exist in multiple crystalline forms with distinct physicochemical properties. The five most well-known polymorphic forms are Polymorphs I, II, III, IV, and V. Polymorph I is the most common form and is typically obtained during industrial production and storage. Polymorph II is metastable and readily converts to Polymorph I upon exposure to moisture or heat, making it less desirable for pharmaceutical applications. Polymorph III is considered a hydrate, containing water molecules within its crystal lattice. Polymorph IV is a rare form, and Polymorph V is a high-pressure form obtained under extreme conditions. Among these, Polymorph I is the most stable, while Polymorph III, the hydrate form, is the most soluble due to the presence of water molecules within its structure, which enhances its dissolution characteristics. Polymorphism in CBZ has significant implications for drug formulation, stability, and bioavailability, and careful consideration of the specific polymorphic form is essential in pharmaceutical development [24].

### 2.2. CBZ Formulation (CBZ-F)

In addition to choosing the best polymorphic form, crystalline habit, and particle size for a formulation in development, excipients can also play a critical role in tablet formulation, ensuring drug stabilization and adequate dissolution [14,15,25]. Some excipients present in CBZ-F, such as magnesium stearate, crospovidone, povidone, croscarmellose sodium, and sodium lauryl sulfate, may potentially impact the disintegration and dissolution profiles of carbamazepine tablets [6,26,27]. However, since the tablets were solubilized in aqueous mixture with surfactant before being administered to the animals, as described earlier, it was not expected that there would be an impact on CBZ-F permeability, since it is already a high-permeability API. The dissolution medium’s pH and the presence of solubilizers (surfactants) or non-aqueous solvents including sodium taurocholate, sodium lauryl sulfate, polysorbate (Tween) 20/80, sodium deoxycholate, cetyltrimethylammonium bromide, lecithin, sodium cholate, sodium oleate, and sodium glycocholate in the dissolution medium often enhance dissolution patterns [28,29].

### 2.3. Neuron Viability Assessment In Vitro

**Figure 5:**
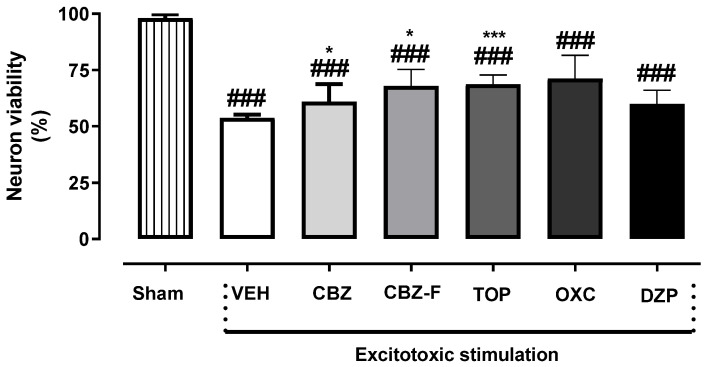
Glutamate- and glycine-induced over-stimulation and excitotoxicity protocol for viability assessment in vitro. Cultured cortical neurons at 37 °C were treated with 10 µM of glutamate/1 µM of glycine for 1 h in the presence of VEH (vehicle), CBZ (carbamazepine), CBZ-F (carbamazepine formulation), TOP (topiramate), OXC (oxcarbazepine), and DZP (diazepam) at 1 mg/mL. The group of untreated cultured neuron without excitotoxic stimulation (sham) was assigned with 100% viability. Data are represented as mean ± SEM, *n* = 10 different cultures. * *p* < 0.05 and *** *p* < 0.001 as compared to the VEH; ### *p* < 0.001 as compared with the sham group (one-way ANOVA followed by Bonferroni’s post hoc test).

One-way ANOVA with post hoc analysis showed significant differences between all comparisons: sham vs. VEH, CBZ, CBZ-F, TOP, OXC, and DZP and VEH vs. CBZ, CBZ-F, and TOP [F(6,63) = 28.81; *p* < 0.0001] as shown in Figure 5. The sham group without stimulation demonstrated no excitotoxic glutamate- and glycine-induced stimulation, unlike stimulated neurons treated with VEH, CBZ, CBZ-F, TOP, OXC, and DZP. In these treatment groups, the excitotoxic stimulation of neurons compromised their viability. The effect of glutamate and glycine were attenuated by CBZ (64.60 ± 3.75; *p* = 0.010), CBZ-F (63.61 ± 3.37; *p* = 0.021) and TOP (72.81 ± 2.78; *p* < 0.0001) at 1 mg/mL when compared to VEH. This finding suggests the restoration of neuron viability by CBZ, CBZ-F, and TOP. The attenuation of glutamate-induced excitotoxic stimulation was associated with the suppression of the release of oxidative stress [30].

### 2.4. In Vivo Assays

#### 2.4.1. Screening for Exploratory and Motor Activities

**Figure 6:**
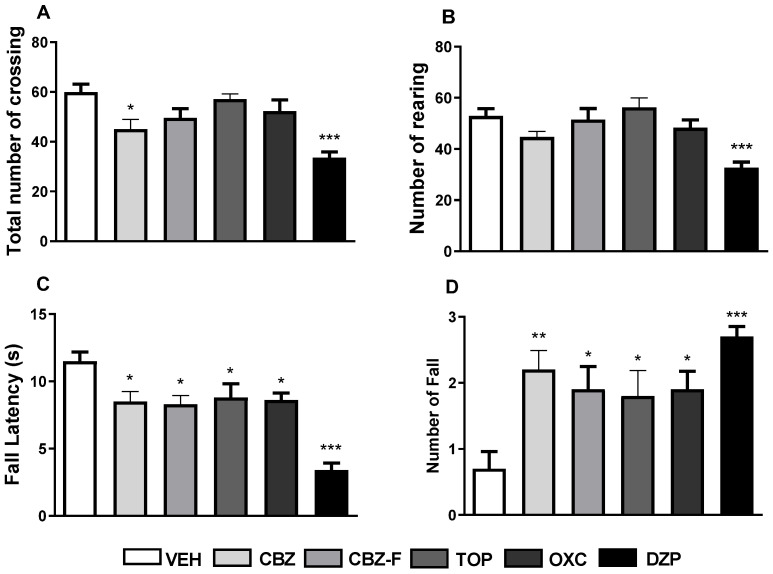
Effect of CBZ-F on the exploratory and motor activities in the open field and rotarod tests, respectively. Oral treatments with VEH (vehicle), CBZ (carbamazepine), CBZ-F (carbamazepine formulation), TOP (topiramate), OXC (oxcarbazepine) and DZP (diazepam), all at the dose of 5 mg/kg, were carried out. The parameters evaluated were total number of crossings (**A**) and number of rearings (**B**) in the open field as well as the fall latency (**C**) and the number of falls (**D**) in the rotarods. Each column represents mean ± S.E.M. (*n* = 10). One-way ANOVA and Dunnett’s post hoc test for multiple comparisons were performed. * *p* < 0.05, ** *p* < 0.01, and *** *p* < 0.001 for other treatment groups vs. vehicle-treated group.

Exploratory and possible anxiolytic-like activity are often assessed in the open field [31]. Mice tend to explore more of the peripheral zone (seemingly safe zone) of the open field. This preference is often associated with an increase in thigmotaxis, crossing, and time spent in the periphery [32,33]. Figure 6 shows the effect of oral treatment with VEH (10 mL/kg) and CBZ, CBZ-F, TOP, OXC, and DZP all at the dose of 5 mg/kg.

One-way ANOVA showed significant differences when comparing the different pharmacological variables [F(5,54 = 7.591; *p* < 0.0001], and the post hoc Dunnett’s test highlighted specific differences between CBZ (45.00 ± 3.90, *p* = 0.0149; Figure 6A) and DZP (33.50 ± 2.41, *p* < 0.0001; Figure 6A), which decreased the total number of crosses when compared to the VEH (59.90 ± 3.17; Figure 6A). In contrast, CBZ-F, TOP, and OXC showed no significant effect on the total number of crossings when compared to the VEH. Furthermore, the DZP group showed a significant effect on the rearing activity (32.70 ± 2.18, *p* < 0.001; Figure 6B) and a decrease in the number of crossings, thereby suggesting the sedation of these animals.

When analyzing fall latency, one-way ANOVA demonstrated significant differences between pharmacological variables [F(5,54) = 13.64; *p* = 0.0001], with the main differences highlighted by the post hoc Dunnett´s test for all variables: CBZ (8.50 ± 0.75; *p* = 0.0185; Figure 6C), CBZ-F (8.30 ± 0.65; *p* = 0.0108; Figure 6C), TOP (8.80 ± 1.01; *p* = 0.0399; Figure 6C), OXC (8.60 ± 0.54; *p* = 0.0241; Figure 6C), and DZP (3.40 ± 0.52; *p* < 0.0001; Figure 6C) compared to the VEH. Figure 6 shows the effect of oral treatment with the VEH (10 mL/kg), and CBZ, CBZ-F, TOP, OXC, and DZP (all at the dose of 5 mg/kg) on the latency for the first fall (C) and the number of falls (D) from the rotarod test.

In addition to these results, one-way ANOVA also demonstrated significant differences in the administration of the different substances [F(5,54) = 4.973; *p* = 0.0008]. Dunnett’s post hoc test highlighted differences for all substances administered: CBZ (2.20 ± 0.29, *p* < 0.0033; Figure 6D), CBZ-F (1.90 ± 0.35, *p* = 0.0248; Figure 6D), TOP (1.80 ± 0.39, *p* = 0.0454; Figure 6D), OXC (1.90 ± 0.28; *p* = 0.0248 Figure 6D), and DZP (2.70 ± 0.15, *p* = 0.0001; Figure 6D) showed significant increases in the number of falls of the animals compared to the VEH. These results indicate that these treatments interfere with the animals’ motor activity up to a dose of 5 mg/kg. The highest frequency of falls of animals treated with DZP, OXC, CBZ, CBZ-F, and TOP suggests a narrow margin (window) of inducing sedation at this dose (5 mg/kg) in comparison to the VEH.

#### 2.4.2. Screening for Barbiturate Sleep Potentiation

**Figure 7:**
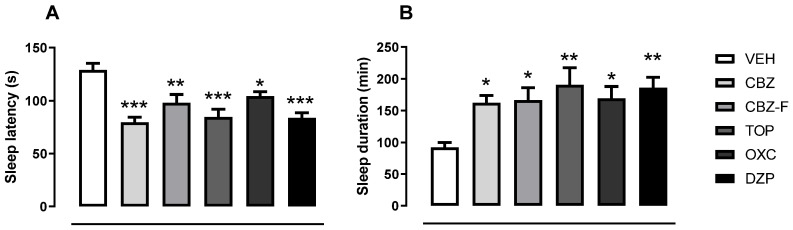
Potentiation and blockade of barbiturate sleep induction. Effect of orally administered vehicle (10 mL/kg), CBZ, CBZ-F, DZP, TOP, and OXC (all 5 mg/kg) on (**A**) sleep latency and (**B**) sleep duration of sodium pentobarbital (40 mg/kg)-induced hypno-sedative effect. * Indicates *p* < 0.05, ** indicates *p* < 0.01 and *** indicates *p* < 0.001 as compared with vehicle-treated group (one-way ANOVA followed by Dunnett’s post hoc test). All groups were treated with sodium pentobarbital (40 mg/kg, i.p.). Results are expressed as mean ± SEM; *n* = 10 in each group. VEH (vehicle), CBZ (carbamazepine), CBZ-F (carbamazepine formulation), TOP (topiramate), OXC (oxcarbazepine), DZP (diazepam), and FLU (flumazenil).

In the barbiturate sleep-induction test, the oral administration of CBZ, CBZ-F, TOP, OXC, and DZP (all at the dose of 5 mg/kg) reduced sleep latency and increased sleep duration significantly when compared to the vehicle (Figure 7A and Figure 7B, respectively) [F(5,54) = 9.358; *p* = 0.0001]. Dunnett’s post hoc test showed significant differences for CBZ (79.60 ± 4.80; *p* < 0.0001), CBZ-F (98.10 ± 7.65; *p* = 0.0031), TOP (84.50 ± 7.49; *p* < 0.0001), OXC (104.2 ± 4.26; *p* = 0.0232), and DZP (83.80 ± 4.85; *p* < 0.0001), which reduced the sleep latency in comparison to the VEH (128.9 ± 6.24). In addition to these results, one-way ANOVA demonstrated significant differences between the variables investigated [F(5,54) = 3.963; *p* = 0,0039], with Dunnett´s post hoc test highlighting specific differences for CBZ (162.4 ± 11.50; *p* = 0.0314), CBZ-F (166.7 ± 19.46; *p* = 0.0202), TOP (190.6 ± 26.99; *p* = 0.0013), OXC (169.5 ± 18.55; *p* = 0.0150), and DZP (185.8 ± 16.77; *p* = 0.0023), which reduced the sleep duration in comparison to the VEH (92.10 ± 7.50). This model was used in a previous evaluation to screen for CNS depressants or stimulants [34]. The effect of barbiturates, which was potentiated, suggests the CNS-depressive activity of these AEDs.

Furthermore, VEH and FLU were combined with different drugs (orally and intraperitoneally administered) to also evaluate outcomes on sleep latency and duration. One-way ANOVA demonstrated significant differences for both dependent variables: sleep latency [F(9,90) = 21.75; *p* = 0.0001] and sleep duration [F(9,90) = 11.02; *p* = 0.0001]. Table 4 demonstrates the differences highlighted by Bonferroni’s post hoc test, showing the comparison between drugs administered orally and intraperitoneally for both the sleep latency and duration (section A and B of Table 4, respectively).

#### 2.4.3. Screening for Antiseizure Activities

**Figure 8:**
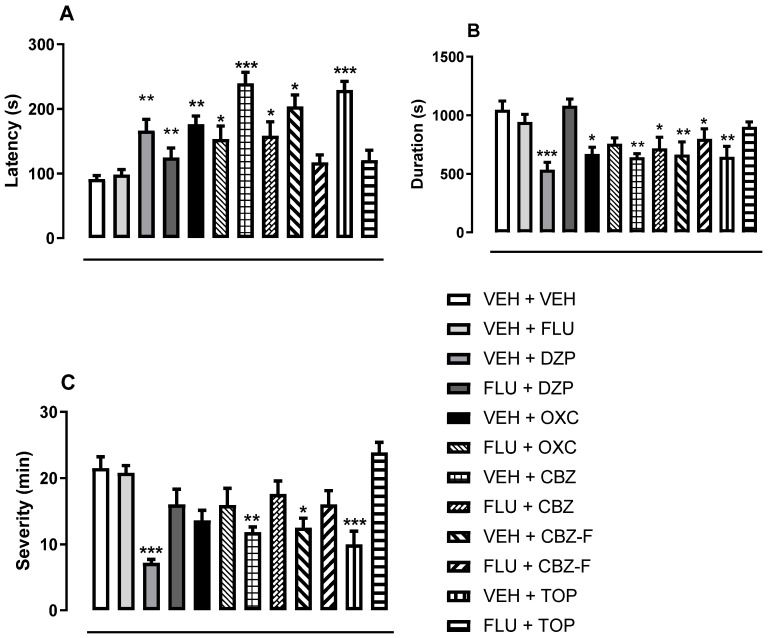
Screening for anticonvulsant activity and its pharmacological blockade using pentylenetetrazol-induced seizure test. The effects of the vehicle (10 mL/kg), CBZ, CBZ-F, TOP, OXC, or DZP at 5 mg/kg on the latency (**A**), duration (**B**), and severity (**C**) of seizure were analyzed by either ANOVA and Dunnettʼs post hoc test with results in mean ± S.E.M. (*n* = 10). Statistical analysis was performed by one-way ANOVA followed by Dunnett’s post hoc test. * *p* < 0.05, ** *p* < 0.01, and *** *p* < 0.001 for other treatment groups vs. VEH group regarding the antiseizure-like effect of CBZ, CBZ-F, TOP, OXC, or DZP. VEH (saline), CBZ (carbamazepine), CBZ-F (carbamazepine formulation), TOP (topiramate), OXC (oxcarbazepine), DZP (diazepam) and FLU (flumazenil).

In the PTZ-induced seizure, the oral administration of CBZ, CBZ-F, TOP, OXC, or DZP at 5 mg/kg exhibited (a) an increase in the latency to convulsion [F (11, 108) = 10.33, *p* < 0.001, ANOVA followed by Dunnett’s post hoc test, Figure 8A] as compared to the VEH group; (b) a reduction in seizure duration [F (11, 108) = 5.85, *p* < 0.001, ANOVA followed by Dunnett’s post hoc test, Figure 8B]; (c) a reduction in the severity of PTZ-induced convulsion [F (11, 108) = 8.09, *p* < 0.001, ANOVA followed by Dunnett’s post hoc test, Figure 8C]. This anticonvulsant-like effect was attenuated significantly by FLU (2 mg/kg—an antagonist of the benzodiazepine site of GABA A), as shown in Figure 8 A–C—the pharmacological blockade of the benzodiazepine site of the GABAA receptor, understood as the possible mechanism of CBZ-F. This finding is consistent with a report implicating the modulation of voltage-gated ion channels, increased inhibitory neurotransmission mediated by gamma-aminobutyric acid (GABA), and/or the attenuation of excitatory neurotransmission mediated by glutamate in the mechanism underlying the anticonvulsant properties [35].

As an inductor of seizure in this study and a noncompetitive antagonist of the GABA A receptor complex [36], PTZ provides mechanistic clues of GABAergic-related pathways in the effects of tested AEDs and justifies the use of flumazenil to block the benzodiazepine site. Currently, the exact mechanism of action of topiramate and carbamazepine is not fully understood. Meanwhile, the choice of the benzodiazepine site does not necessarily exclude the involvement of other targets. In a PTZ-induced seizure, the possible enhancement of GABAergic transmission by AEDs and the blockade of their anti-seizure property with agents capable of blocking the allosteric binding site of GABA A receptors was considered important at this preliminary stage of our study. The AEDs and PTZ may have elicited opposite effects on GABA receptor currents as a result of their actions on the GABA A receptor complex. Previous data reported that topiramate enhances GABA-mediated chloride flux and GABA-evoked chloride currents in murine brain neurons and increases seizure threshold [37]. As the CBZ main target-voltage-gated sodium channels are reported elsewhere [21], the assessment of GABAergic involvement in this study expands the mechanistic discussion around CBZ and other AED-induced increases in the seizure threshold or propagation, which in the hypothesis is more likely to affect neural excitability.

## 3. Materials and Methods

### 3.1. Methods

CBZ and CBZ-F were kindly gifted by Teuto Ltd. (Anápolis, Brazil). Pentylenetetrazol (PTZ), topiramate (TOP), oxcarbamazepine (OXC), diazepam (DZP) and flumazenil (FLU) were obtained from Sigma Aldrich, St. Louis, MO, USA. Chemicals used in the current study were of analytical grade without further purification.

### 3.2. Carbamazepine (CBZ)

#### 3.2.1. Computational Procedures

In the literature, we found five different forms (polymorphs) for carbamazepine: Form I [10], Form II [38], Form III [39], Form IV [40], and Form V [41]. The crystallographic data for these forms were obtained from the Cambridge Crystallographic Data Centre (CCDC) under the following codes: 185919, 1121423, 814268, 249934, and 791775, respectively. The analysis of molecular interactions, structural comparison, and molecular representation was performed using the Mercury [42], Olex2 [43], and CrystalExplorer software (version 17.5). The molecular interactions can be described by geometric parameters and further enriched based on electronic density through Hirshfeld surfaces (HSs), as well as quantified using 2D fingerprint plots [44,45,46,47]. The HSs were generated considering the molecule’s density and the distribution neighborhood pattern, based on the distance from the surface to the nearest atom in the molecule itself (de) and the distance from the surface to the nearest atom in another molecule (di) [45,46]. The 2D fingerprint plots are generated by plotting de versus di distribution, quantifying the contacts present in the analyzed molecules [47].

Additionally, the theoretical analysis for CBZ forms was performed using the density functional theory [48]. The geometric conformations of the five molecules were optimized in the gas phase to analyze some parameters with greater conformational freedom. The optimization was carried out using the Gaussian09 software (version 7.0), employing the M06-2X/6-311++G (d,p) theory level [49,50,51] which is recommended for noncovalent interactions and considers electronic correlation [49,52,53,54]. The wave function generated in this calculation was then used to calculate the frontier molecular orbitals (FMOs) and the molecular electrostatic potential (MEP) map, allowing the identification of orbitals associated with electrophilic/nucleophilic behavior and regions with the highest reactivity sites, respectively [55,56,57].

#### 3.2.2. Preparation of CBZ-F

First, 200 mg CBZ tablets were prepared, with the following excipients: magnesium stearate, microcrystalline cellulose, crospovidone, povidone, croscarmellose sodium, colloidal silicon dioxide, and sodium lauryl sulfate. The tablet manipulation process, as well as its exact quantitative composition, cannot be disclosed, as they are intellectual property of Teuto Brazilian Laboratory S/A. The CBZ-F excipients, which vary, are [magnesium stearate (lubrificant), microcrystalline cellulose (diluent), crospovidone (disintegrant), povidone (binder), croscarmellose sodium (disintegrant), colloidal silicon dioxide (glidant), sodium lauryl sulfate (anionic tensoative)] [1]. The concentration of these excipient varies from 0.1% of colloidal silicon dioxide to the maximum 90% of microcrystalline cellulose.

### 3.3. Pharmacological Approaches

#### 3.3.1. Excitotoxicity Protocol and Neuron Viability Assessment In Vitro

Mice cortical neurons were harvested and digested before the suspension of the neuronal pellets in growth media that consisted of penicillin (10 units/mL), 2% of both NuSerum and B27, streptomycin (10 µg/mL), and L glutamine (29.2 µg/mL) seeded on poly-D-lysine (50 µg/mL)-coated coverslips. Forty-eight hours after plating, cultures were treated with uridine and 5-fluoro-2′-deoxyuridine (at 3.5 mg/mL and 1.5 µg/mL, respectively) to kill active cells. Half of the conditioned medium was replaced with fresh medium while maintaining 3–4 days of neuronal feeding. At 37 °C, 10 µM of glutamate/1 µM of glycine per 1 h was added to stimulate cultured neurons. Conditioned media were replaced by half with fresh growth media containing 2× glutamate/glycine. The CBZ, CBZ-F, TOP, or OXC 1 mg/mL were diluted in the stimulation media. Viability of 100% was assigned to the sham group (cultured neurons without excitotoxic stimulation and treatment). After stimulation, cultures were incubated at 37 °C for 24 h before viability assessment through the metabolic activity assessed with MTS (3-(4,5-dimethylthiazol-2-yl)-5-(3-carboxymethoxyphenyl)-2-(4-sulfophenyl)-2H-tetrazolium) treatment. Both cytotoxic and viable cells represent the total cell number. The viability and cytotoxic nuclei thresholds were set at >50 µm^2^ and >20 µm^2^, respectively.

#### 3.3.2. Animals and Treatment

In vivo assays were conducted with Swiss albino mice weighing approximately 25–30 g (6–8 weeks old) provided by the Central Bioterium of the Evangelical University of Goiás. Ten animals were housed per bioterium cage (320 × 180 × 160 cm) with free access to water and food under controlled temperature and light (22 ± 2 °C, 12 h light/dark cycle). The Ethics Committee for the Use of Animals from the Evangelical University of Goiás-Brazil approved all experimental protocols (nº 022/19). Experiments were conducted between 1200 and 1800 h in the apparatus with an illumination level of approximately 40 lux. The studied compounds (CBZ, CBZ-F, TOP, OXC) in tablet form were crushed and suspended in an aqueous solution-containing surfactant—0.5% Tween 80 (vehicle—VEH)—like PTZ, which was already in powdery form from the supplier. The addition of this surfactant improves the dissolution rate and bioavailability of hydrophobic drugs. The DZP and FLU in commercial solution were diluted in this vehicle into an appropriate concentration before oral administration. The oral drug dose of 5 mg/kg extrapolated from pilot experiments (unpublished data) provides the basis for a dose variability-free comparison of biological effects. Control group animals received 10 mL of VEH per kg of body weight (10 mL/kg).

#### 3.3.3. Screening for Exploratory and Motor Activities

Both an open field and rotarod were used to evaluate alterations in exploratory and motor activities. The open field is a conventional model for assessing exploratory activities in laboratory mice. The circular open field apparatus with the illumination level of approximately 40 lux consisting of an acrylic arena 20 cm high and 36 cm in diameter, with its surface divided into eight equal sectors [58], was used to evaluate the exploratory activity of CBZ-F. The animals were treated orally with VEH (10 mL/kg), as well as CBZ, CBZ-F, TOP, OXC, and DZP, all at the dose of 5 mg/kg, before their individual placement in the center of the open field arena, and they were recorded for 5 min. The number of crossings between sectors and rearings were evaluated according to the previous studies [59,60,61]. The motor coordination of rodents is widely assessed in a rotarod [62]. This apparatus is a rotating (at a constant speed—12 rpm) non-slippery iron bar platform which is 3 cm in diameter and 30 cm in length. All animals were introduced to the rotarod for 2 min to acclimate and screen for motor deficits for inclusion or exclusion 24 h prior to the administration of drugs and re-exposure during a 1 min single-test session. After 24 h of acclimatization and screening, VEH (10 mL/kg), as well as CBZ, CBZ-F, TOP, OXC, and DZP (all at the dose of 5 mg/kg), were orally administered prior to testing (60 min interval). According to the previous studies [58,63,64,65], the latency period for the first fall and the number of falls were analyzed.

#### 3.3.4. Barbiturate Sleep Induction

Possible CNS depression or stimulation was evaluated using a barbiturate sleep potentiation strategy. The barbiturate sleep induction was carried out essentially as previously described [59,61,66]. The VEH (10 mL/kg), CBZ, CBZ-F, TOP, OXC, or DZP at 5 mg/kg was orally administered 1 h before sodium pentobarbital (40 mg/kg, intraperitoneal—i.p.) at a 1 h interval. The sleep latency, measuring the time taken for the loss of the righting reflex and sleep duration for the voluntary recovery of the righting reflex of mice (*n* = 10), was analyzed to detect CNS depression or stimulation. Possible GABAergic mechanisms in the CNS-like activities were investigated through the intraperitoneal pretreatment of the animals with the VEH (10 mL/kg) or flumazenil (FLU 2 mg/kg, a competitive antagonist of the benzodiazepine site of the GABAA receptor). After 30 min of this pretreatment, the animals were treated orally with the VEH (10 mL/kg), CBZ, CBZ-F, TOP, OXC, and DZP at 5 mg/kg. Sixty minutes after the oral treatment, all groups of mice were treated accordingly and were posteriorly treated with sodium pentobarbital (40 mg/kg, i.p.) to record the sleep latency and duration (Table 5).

#### 3.3.5. Screening for Anticonvulsant Activity and Its Pharmacological Blockade

PTZ-induced seizure in mice was employed to evaluate the antiseizure effect of CBZ-F according to a previous study [61]. Mice (*n* = 10) were randomly divided into treatment groups receiving an oral administration of the VEH, CBZ, CBZ-F, TOP, OXC, or DZP at 5 mg/kg. PTZ is considered a noncompetitive antagonist of the GABA A receptor; PTZ could reduce GABAergic transmission [36]. After 60 min of oral administration, each mouse was subjected to an intraperitoneal injection of PTZ solution at a dose of 70 mg/kg. The mice were subsequently observed for the onset time of myoclonic jerks and the onset time and duration of seizures, while the severity of the seizure was measured with collective changes in mice behavior (myoclonic jerks, vocalization, Straub, akinesia, tremor, leap, paralysis, chronic seizure, rigidity, and tonic extension of the hind limbs with death). The mouse mortality rate (%) was calculated for each group using the following formula: [(N − nd)/N]100, where N indicates total number of animals and nd is the number of deaths, as described in a previous experiment [59,61,67]. To investigate the possible underlying mechanisms of the anticonvulsant effect, mice were pretreated intraperitoneally with saline substance (VEH) (10 mL/kg) or FLU (2 mg/kg) (an antagonist of the benzodiazepine site of GABA A) before (30 min interval) the oral administration of the VEH (10 mL/kg), CBZ, CBZ-F, TOP, OXC, or DZP at 5 mg/kg (Table 1). The latency, duration, and severity of seizures were analyzed after the induction of convulsion with PTZ injection. The arrangement between procedures was compared between each drug combination.

### 3.4. Statistical Analysis

After analyzing data normality assumptions, a one-way ANOVA was performed to compare the dependent variables and Dunnett´s test determined the effects of the administered drugs compared to the VEH-treated group (control). The experimental set-up with FLU pretreatment and treatment was analyzed (ANOVA and Bonferroni multiple comparison test). Grubb’s test at the alpha value of 5% set was used to detect the possible occurrence of outliers in each data set. Results are expressed as mean ± S.E.M and considered statistically significant at *p* values < 0.05.

## 4. Conclusions

CBZ polymorphs are stabilized by C-H…O and N-H…O interactions, with the presence of a dimeric interaction in Forms I–IV, which in solid state is related to the donation–reception of electrons between the amide groups which form the N-H…O interaction. For these CBZ polymorphs, the theoretical analysis suggests that the oxygen atom of the amide group is more susceptible to electrophilic attacks. The CBZ-F and the reference AEDs potentiated barbiturate sleep, altered motor activities, and attenuated PTZ-induced convulsion. However, flumazenil pretreatment blocked these effects. As research that is relevant to epilepsy therapy development is gaining attention from a new formulation’s perspective, additional preclinical assessments could further establish the promising utility of CBZ-F in clinical settings while expanding the scope of AED formulation design for optimization. This preliminary screening will benefit from future extensive pharmacodynamic and pharmacokinetics profiling that focuses on the spectrum of therapeutic and adverse effects.

## Figures and Tables

**Table 1:** Crystallographic parameters for CBZ crystalline forms.

Parameters		Form I	Form II	Form III	Form IV	Form V
Chemical formula		C_15_H_12_N_2_O	C_15_H_12_N_2_O	C_15_H_12_N_2_O	C_15_H_12_N_2_O	C_15_H_12_N_2_O
Crystal system		Triclinic	Trigonal	Monoclinic	Monoclinic	Orthorhombic
Space group		P$\bar{1}$	R$\bar{3}$	P2_1_/n	C2/c	P*bca*
Z, Z^′^		8, 4	18, 1	4, 1	8, 1	8, 1
Unit cell parameters	a (Å)	5.1705 (6)	35.454 (3)	7.5500 (16)	26.609 (4)	9.1245 (5)
	b (Å)	20.574 (2)	35.454 (3)	11.186 (3)	6.9269 (10)	10.4518 (5)
	c (Å)	22.245 (2)	5.253 (1)	13.954 (3)	13.957 (2)	24.8224 (11)
	α (°)	84.124 (4)	90	90.00	90.00	90.00
	β (°)	88.008 (4)	90	92.938 (8)	109.702 (2)	90.00
	γ (°)	85.187 (4)	120	90.00	90.00	90.00
Volume cell (Å^3^)		2344.8 (5)	5718.32	1176.9 (5)	2421.9 (6)	2367.2 (2)

**Table 2:** Bond lengths and angles for the interaction (N-H…O dimer) present in CBZ crystalline forms.

Crystalline Form	d (N-H) (Å)	d (H...O) (Å)	d (N...O) (Å)	d (N-H…O) (°)
Form I	0.908	1.978	2.864	176.93
Form II	-	-	2.890	-
Form III	0.862	2.075	2.937	176.77
Form IV	0.928	1.943	2.847	177.55
Form V	-	-	-	-

**Table 3:** Percentage of interactions present in CBZ forms.

Crystalline Form	H…H	C…H	O…H	C…C	Others
Form I	47.5%	34.3%	13.7%	1.8%	2.7%
Form II	-	-	-	-	-
Form III	53.7%	22.6%	13.3%	8.2%	2.2%
Form IV	50.6%	29.5%	13.2%	4.6%	2.7%
Form V	48%	35.7%	13.%	0.8%	2.5%

**Table 4:** Significant differences highlighted by Bonferroni’s post hoc test between drugs administered orally and intraperitoneally for the variables of both sleep latency (Table A) and sleep duration (Table B).

Table A	VEH + VEH	VEH + FLU	VEH + OXC	FLU + OXC	VEH + CBZ	FLU + CBZ	VEH + CBZ-F	FLU + CBZ-F	VEH + TOP	FLU + TOP
VEH + VEH	-	*p* = 0.999	*p* = 0.300	*p* = 0.001	*p* = 0.0001	*p* = 0.0001	*p* = 0.0001	*p* = 0.0001	*p* = 0.0001	*p* = 0.011
VEH + FLU		-	*p* = 0.999	*p* = 0.021	*p* = 0.0001	*p* = 0.0001	*p* = 0.0001	*p* = 0.0001	*p* = 0.0001	*p* = 0.107
VEH + OXC			-	*p* = 0.999	*p* = 0.0008	*p* = 0.173	*p* = 0.0001	*p* = 0.0001	*p* = 0.0041	*p* = 0.999
FLU + OXC				-	*p* = 0.1532	*p* = 0.999	*p* = 0.0001	*p* = 0.0098	*p* = 0.5386	*p* = 0.999
VEH + CBZ					-	*p* = 0.999	*p* = 0.1737	*p* = 0.999	*p* = 0.999	*p* = 0.0311
FLU + CBZ						-	*p* = 0.0008	*p* = 0.7944	*p* = 0.999	*p* = 0.999
VEH + CBZ-F							-	*p* = 0.999	*p* = 0.0439	*p* = 0.0001
FLU + CBZ-F								-	*p* = 0.999	*p* = 0.0016
VEH + TOP									-	*p* = 0.1267
FLU + TOP										-
**Table B**										
VEH + VEH	-	*p* = 0.999	*p* = 0.0118	*p* = 0.191	*p* = 0.051	*p* = 0.2994	*p* = 0.0001	*p* = 0.0003	*p* = 0.0001	*p* = 0.999
VEH + FLU		-	*p* = 0.0240	*p* = 0.353	*p* = 0.0984	*p* = 0.540	*p* = 0.0001	*p* = 0.0008	*p* = 0.0001	*p* = 0.999
VEH + OXC			-	*p* = 0.999	*p* = 0.999	*p* = 0.999	*p* = 0.999	*p* = 0.999	*p* = 0.0073	*p* = 0.999
FLU + OXC				-	*p* = 0.999	*p* = 0.999	*p* = 0.424	*p* = 0.999	*p* = 0.0003	*p* = 0.999
VEH + CBZ					-	*p* = 0.999	*p* = 0.999	*p* = 0.999	*p* = 0.0015	*p* = 0.999
FLU + CBZ						-	*p* = 0.275	*p* = 0.999	*p* = 0.0001	*p* = 0.999
VEH + CBZ-F							-	*p* = 0.999	*p* = 0.999	*p* = 0.0314
FLU + CBZ-F								-	*p* = 0.162	*p* = 0.399
VEH + TOP									-	*p* = 0.0001
FLU + TOP										-

Results are expressed as *p* value; *n* = 10 in each group. VEH (vehicle), CBZ (carbamazepine), CBZ-F (carbamazepine formulation), TOP (topiramate), OXC (oxcarbazepine), DZP (diazepam), and FLU (flumazenil). The values in bold represent where the Bonferroni post hoc test indicated significant differences between the drug combination.

**Table 5:** Treatment groups, doses, and administration routes.

Group	Pharmacological Doses and Routes
1	VEH (10 mL/kg, i.p) + VEH (10 mL/kg, p.o)
2	VEH (10 mL/kg, i.p) + CBZ (5 mg/kg, p.o)
3	FLU (2 mg/kg, i.p) + CBZ (5 mg/kg, p.o)
4	VEH (10 mL/kg, i.p) + FLU (2 mg/kg, p.o)
5	VEH (10 mL/kg, i.p) + OXC (5 mg/kg, p.o)
6	FLU (2 mg/kg, i.p) + OXC (5 mg/kg, p.o)
7	VEH (10 mL/kg, i.p) + CBZ-F (5 mg/kg, p.o)
8	FLU (2 mg/kg, i.p) + CBZ-F (5 mg/kg, p.o)
9	VEH (10 mL/kg, i.p) + TOP (5 mg/kg, p.o)
10	FLU (2 mg/kg, i.p) + TOP (5 mg/kg, p.o)

Subtitle: VEH—vehicle; CBZ—carbamazepine; CBZ-F—carbamazepine formulation; TOP—topiramate; OXC—oxcarbazepine; DZP—diazepam; FLU—flumazenil; i.p—intraperitoneal; p.o—oral administration.

## Data Availability

Data is contained within the article.
